# Supplementary material for: The genome sequence of Hoary Whitlowgrass, Draba incana L
Source: Wellcome Open Res. 2024 Oct 29;9:630. [Version 1] doi: 10.12688/wellcomeopenres.23202.1 (PMC11612554; doi:10.12688/wellcomeopenres.23202.1)
Supplement: Peer review history

# Reviewer report: Reviewer response for version 1

**DOI:** 10.21956/wellcomeopenres.25552.r108956

**Published:** 2024-12-02

**Authors:** Satish Maurya

**Affiliations:** 1 Bajaj College of Science, Wardha, Maharashtra, India; 2 Agharkar Research Institute, Pune, India

The manuscript is well written, along with the methods and results. All fine details are described well and fit the scope of this journal.

 The manuscript reports the whole genome of *Draba incana *and would be helpful in future studies on the genus or families *.*

**Just an opinion:** However, it would have added more value to the manuscript if the author had added some rigorous analysis and comparisons with other available genomes. 

 I strongly recommend this manuscript be accepted in this format for indexing.

Are sufficient details of methods and materials provided to allow replication by others?

Yes

Is the rationale for creating the dataset(s) clearly described?

Yes

Are the datasets clearly presented in a useable and accessible format?

Yes

Are the protocols appropriate and is the work technically sound?

Yes

Reviewer Expertise:

Genomics, Phylogenomics, Evolution, Biogeography, NGS

I confirm that I have read this submission and believe that I have an appropriate level of expertise to confirm that it is of an acceptable scientific standard.

# Reviewer report: Reviewer response for version 1

**DOI:** 10.21956/wellcomeopenres.25552.r108962

**Published:** 2024-11-27

**Authors:** Martin A Lysak

**Affiliations:** 1 Masaryk University, Brno, Czech Republic

**Keywords:** what is "chromosomal"? I propose to include "polyploidy" and  "Arabideae" (included in Species taxonomy)

**Background:**

 It seems to be a little strange to refer to the paper from 1987 if there are at least two papers by Jordon-Thaden and later papers on the tribe Arabideae from the same group (M. Koch).

 The authors used PacBio HiFi long-read sequencing in combination with chromosome conformation Hi-C data. The final assembly has a total length of 667.80 Mb in 474 scaffolds (with 1163 gaps).

 Error in fold coverage for HiFi data?

 43.53 Gb / 667.80 Mb = 65.18-fold change, not 126-fold change

 (Hi-C data: 152.91 Gb / 667.80 = 229-fold change)

 A typing error in ‘Stupiké polní rané’. Should be ‘Stupické polní rané‘.

 The note on D. incana being an allotetraploid species is not clear. What is the evidence? How the genome was phase? I could only locate that " While not fully phased, the assembly deposited is of one haplotype"

 As the information on D. incana being allo- or autotetraploid species is imoportant and can be (erroneously) adopted by other authors, I consider this issue the most important point to resolve before the final indexing of the paper.

 Do authors consider using SubPaser ( https://github.com/zhangrengang/SubPhaser) to phase subgenomes?

Are sufficient details of methods and materials provided to allow replication by others?

Partly

Is the rationale for creating the dataset(s) clearly described?

Yes

Are the datasets clearly presented in a useable and accessible format?

Yes

Are the protocols appropriate and is the work technically sound?

Yes

Reviewer Expertise:

Martin Lysak - comparative and evolutionary plant genomics, particularly the crucifers

I confirm that I have read this submission and believe that I have an appropriate level of expertise to confirm that it is of an acceptable scientific standard.
